# A Flat Reconstruction of the Medial Collateral Ligament and Anteromedial Structures Restores Native Knee Kinematics: A Biomechanical Robotic Investigation

**DOI:** 10.1177/03635465241280984

**Published:** 2024-10-03

**Authors:** Adrian Deichsel, Christian Peez, Michael J. Raschke, Alina Albert, Mirco Herbort, Christoph Kittl, Christian Fink, Elmar Herbst

**Affiliations:** † Department of Trauma, Hand and Reconstructive Surgery, University Hospital Münster, Münster, Germany; ‡ OCM Clinic, Munich, Germany; § Gelenkpunkt, Sports and Joint Surgery Innsbruck, Innsbruck, Austria; Investigation performed at University Hospital Münster, Münster, Germany

**Keywords:** medial collateral ligament, MCL, anteromedial, rotational instability, Slocum test

## Abstract

**Background:**

Injuries of the superficial medial collateral ligament (sMCL) and anteromedial structures of the knee result in excess valgus rotation and external tibial rotation (ER) as well as tibial translation.

**Purpose:**

To evaluate a flat reconstruction of the sMCL and anteromedial structures in restoring knee kinematics in the combined MCL- and anteromedial-deficient knee.

**Study Design:**

Controlled laboratory study.

**Methods:**

Eight cadaveric knee specimens were tested in a 6 degrees of freedom robotic test setup. Force-controlled clinical laxity tests were performed with 200 N of axial compression in 0°, 30°, 60°, and 90° of flexion: 8 N·m valgus torque, 5 N·m ER torque, 89 N anterior tibial translation (ATT) force, and an anteromedial drawer test consisting of 89 N ATT force under 5 N·m ER torque. After determining the native knee kinematics, we transected the sMCL, followed by the deep medial collateral ligament (dMCL). Subsequently, a flat reconstruction of the sMCL with anteromedial limb, mimicking the function of the anteromedial corner, was performed. Mixed linear models were used for statistical analysis (*P* < .05).

**Results:**

Cutting of the sMCL led to statistically significant increases in laxity regarding valgus rotation, ER, and anteromedial translation in all tested flexion angles (*P* < .05). ATT was significantly increased in all flexion angles but not at 60° after cutting of the sMCL. A combined instability of the sMCL and dMCL led to further increased knee laxity in all tested kinematics and flexion angles (*P* < .05). After reconstruction, the knee kinematics were not significantly different from those of the native state.

**Conclusion:**

Insufficiency of the sMCL and dMCL led to excess valgus rotation, ER, ATT, and anteromedial tibial translation. A combined flat reconstruction of the sMCL and the anteromedial aspect restored this excess laxity to values not significantly different from those of the native knee.

**Clinical Relevance:**

The presented reconstruction might lead to favorable results for patients with MCL and anteromedial injuries with an anteromedial rotatory knee instability.

Injuries to the medial side of the knee occur frequently in conjunction with tears of the anterior cruciate ligament (ACL).^10,29,41^ Although most medial-sided injuries qualify for nonoperative treatment, persistent instability may be present in a subset of patients.^23^ This can increase the stress on an ACL reconstruction, possibly leading to a higher risk of ACL reconstruction failure.^2,3,28,30^

Besides the superficial medial collateral ligament (sMCL), the anteromedial corner of the knee has recently gained interest as an important peripheral stabilizer of the knee. It consists of the deep medial collateral ligament (dMCL), anteromedial capsule (AMC), and anteromedial retinaculum (Figure 1).^36,43,48^ In cases of an ACL tear, injuries to the anteromedial corner were found to be present in up to 72.5% of patients.^20,47^ While anteromedial corner injuries were insufficiently described in previous clinical studies, recent biomechanical studies have described the importance of the anteromedial knee structures, which contribute up to 23% to restrain valgus rotation, external tibial rotation (ER), and tibial translation.^4,22,37,46^ Insufficiency of these structures may result in an anteromedial rotatory instability (AMRI), first described by Slocum and Larson in 1968 as a rotatory and translational instability when the anterior drawer test was performed with an externally rotated foot.^39,40^

**Figure 1.**
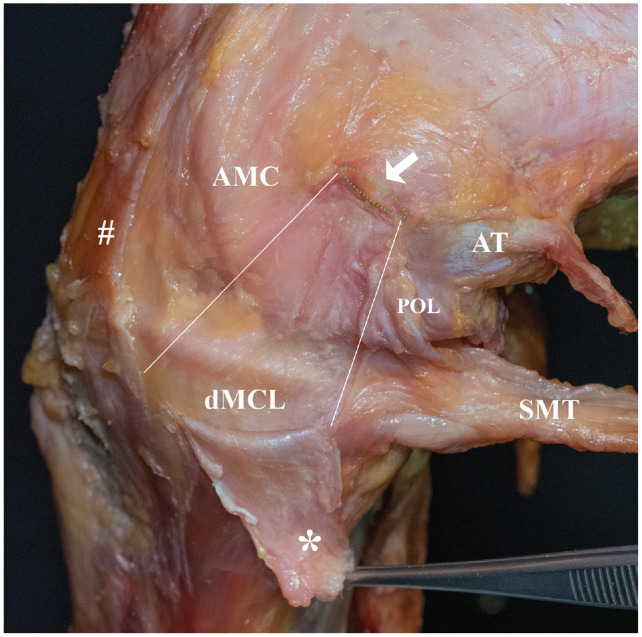
Anatomy of the medial side of the knee. With the knee in 90° of flexion and in external rotation, the superficial medial collateral ligament (sMCL; *) was detached at its femoral longitudinal insertion side (green dashed line) and deflected downward. The center of the medial femoral epicondyle is marked by the white arrow. Below the sMCL, the deep medial collateral ligament (dMCL) is visualized as a triangular structure, tense under external rotation. Anterior to the dMCL lies the anteromedial capsule (AMC). The anteromedial retinaculum (#), which lies above the anteromedial capsule and anterior to the sMCL, was partially resected. Posterior to the dMCL lies the posterior oblique ligament (POL), which is relaxed in flexion. White lines mark the borders between AMC, dMCL, and POL. AT, adductor tubercle; SMT, semimembranosus tendon.

In cases of AMRI, an isolated reconstruction of the sMCL was found to be insufficient to restore knee kinematics to that of the native state.^8,32^ Thus, combined reconstructions of the sMCL and anteromedial structures have been proposed to control AMRI. However, multiple techniques exist for combined reconstruction, and uncertainty exists as to which techniques are able to restore the native knee kinematics.^7-9,26,27,45^

The purpose of this study was to investigate the influence of insufficiencies of the sMCL, dMCL, and AMC on knee kinematics, and to evaluate a novel reconstruction of the sMCL and anteromedial corner utilizing a flat semitendinosus graft. It was first hypothesized that insufficiencies of the sMCL and anteromedial structures lead to increased valgus rotation and ER as well as AMRI. It was further hypothesized that the presented surgical technique of flat sMCL and anteromedial reconstruction is able to restore knee kinematics.

## Methods

Eight unpaired fresh-frozen cadaveric knee specimens (mean age, 70.1 ± 9.5 years; 5 male, 3 female) without previous knee injury, surgery, or high-grade osteoarthritis were obtained from MedCure. The study was performed with permission from the institutional review board of the University of Münster (reference No. 2020-181-f-S).

Specimens were stored at –20°C and thawed for 24 hours at room temperature before preparation. The skin and subcutaneous tissues were resected, leaving fascia and muscles intact. The sartorius fascia and hamstring tendons were resected from their tibial insertion, leaving the anteromedial retinaculum intact. The tibia and femur were cut 200 mm above and below the joint line and secured in aluminum cylinders with 3-component polyurethane resin bone cement (RenCast; Gößl & Pfaff). The fibula was then cut 100 mm distal to the proximal tibiofibular joint and transfixed with a 3.5-mm cortical screw to the tibia.^37^ Specimens were wrapped in tissue paper soaked with water to prevent drying.

### Robotic Test Setup

A validated setup consisting of a 6 degrees of freedom industrial robot (KR 60-3; KUKA Robotics) equipped with a force-torque sensor (FTI Theta; ATI Industrial Automation) was used for biomechanical testing in this study.^8,22,44^ The robotic system allows for displacement-controlled positioning with a repeatability of ±0.06 mm. The force-torque sensor allows for a precision of ±0.25 N and ±0.05 N·m in the force-controlled positioning. Using the custom software simVITRO (Cleveland Clinic BioRobotics Laboratory), we optimized the test system for the simulation and acquisition of knee joint movements. A tactile measuring arm (Absolute Arm 8320-7; Hexagon Metrology GmbH) with an accuracy of 0.05 mm was utilized to define landmarks on the distal femur, tibia plateau and the shaft of the femur and tibia from which a modified Grood and Suntay coordinate system was defined.^19,35^ Data acquisition was performed with a sampling rate of 500 Hz.

Each specimen was preconditioned by flexing and extending the knee 10 times.^31^ After neutralizing all forces and torques acting on the knee in full extension, the passive path was determined by flexing each knee from full extension to 90° of flexion, while minimizing forces (<1 N) and torques (0.1 N·m) in all axes aside from the flexion-extension axis. An axial compression force of 50 N was applied to keep the femur and tibia in contact during the passive path. For determination of the knee kinematics, a force-controlled testing protocol was performed, meaning that displacements in response to given forces/torques were recorded. At 0°, 30°, 60°, and 90° of flexion, the following test protocols were performed under axial compression of 200 N (simulating partial weightbearing during rehabilitation): 8 N·m valgus angulation, 5 N·m internal tibial rotation torque, 5 N·m ER torque, 89 N anterior tibial translation (ATT) force, and 89 N ATT force under 5 N·m ER torque, simulating the AMRI test (Slocum test, presented in millimeters of ATT; referred to as anteromedial translation in the following text).^13,40^

### Sequential Cutting and Reconstruction Protocol

**Figure 2.**
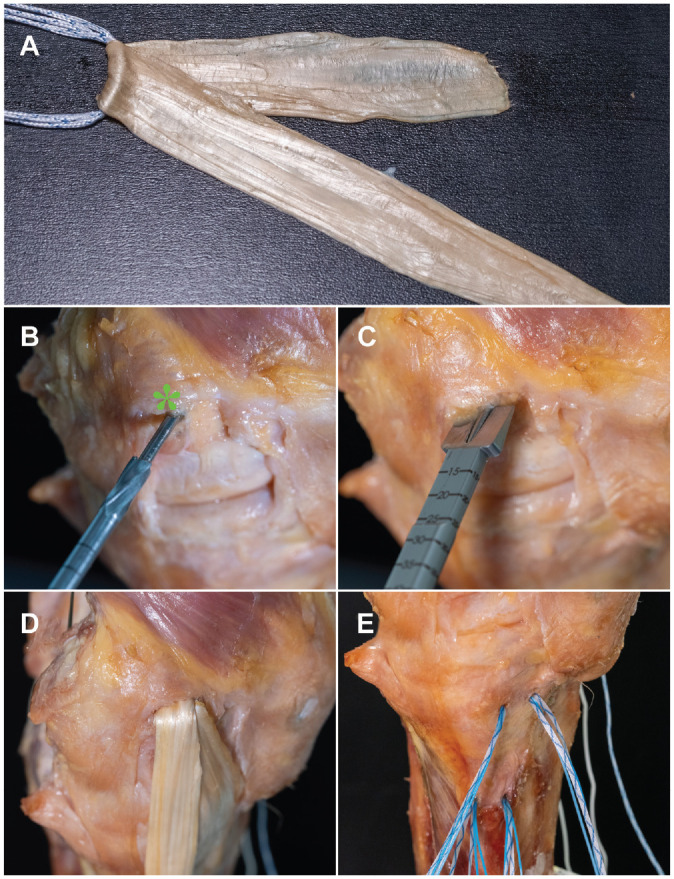
Flat reconstruction of the superficial medial collateral ligament (sMCL) with anteromedial limb in a right knee. The right side in the picture corresponds to anterior in the knee. (A) Flattened semitendinosus tendon is doubled over an adjustable loop button to form a graft with 2 limbs for reconstruction of the sMCL and anteromedial limb. (B) A 2.0-mm K-wire is placed in the center of the medial epicondyle (asterisk) and overdrilled with a cannulated 4.5-mm drill. (C) A flat femoral tunnel is created with a cannulated chisel the size of the graft. The femoral tunnel is placed horizontal with the tibial plateau with the knee in 20° of flexion. (D) The adjustable loop button is shuttled through the 4.5-mm bone tunnel and the graft pulled into the flat tunnel. (E) Four suture anchors are placed in the attachment areas of the sMCL and deep medial collateral ligament for tibial fixation.

**Figure 3.**
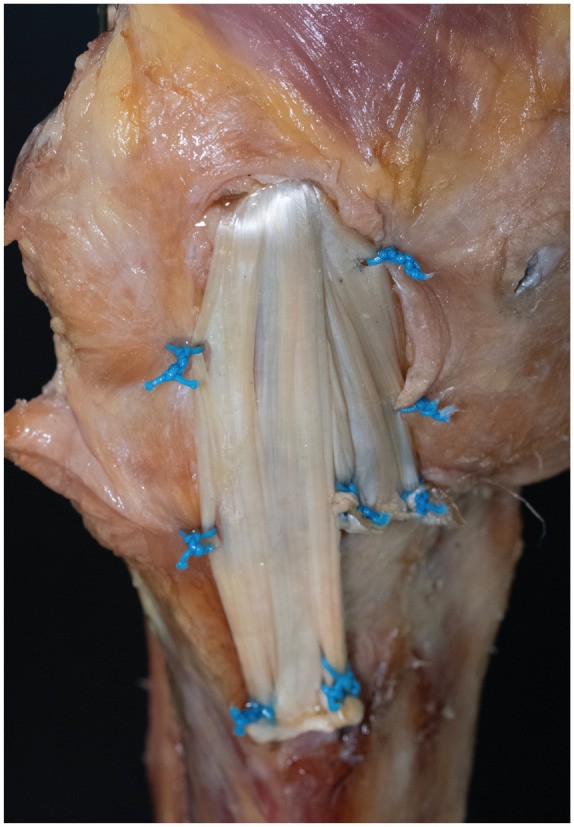
Flat reconstruction of the superficial medial collateral ligament (sMCL) with anteromedial limb in a right knee. The right side in the picture corresponds to anterior in the knee. The reconstruction was sutured to the posterior oblique ligament and anteromedial retinaculum using No. 2 sutures.

After acquiring the native knee joint kinematics, the sMCL was released from its tibial insertion and resected over its full length, while keeping the dMCL and AMC intact. In the following step, the dMCL and overlying anteromedial retinaculum were resected. The flat reconstruction of the sMCL and anteromedial corner was performed according to a previously described technique, with slight modifications (use of cannulated chisels and creation of bone tunnels in 20° of flexion).^1^ First, the previously harvested semitendinosus tendon was partially incised longitudinally and flattened using a raspatorium to produce a flat graft.^16^ The length of the graft was trimmed to 24 cm. The flattened tendon was doubled over the loop of an adjustable cortical button (FairFix; Medacta), so that one-third of the length was available for the anteromedial limb and two-thirds for the sMCL reconstruction. After sizing of the flat graft, a 2.0-mm K-wire was drilled through the center of the medial femoral epicondyle and overdrilled with a 4.5-mm cannulated drill. Next, a flat femoral bone tunnel was created using a cannulated chisel (Medacta) based on the graft size to a depth of 20 mm (Figure 2). The femoral bone socket was oriented parallel to the joint line in 20° of flexion, to best simulate the angulation of the femoral attachment of the sMCL.^36^ The adjustable button was shuttled through the 4.5-mm tunnel, and the graft was pulled into the femoral tunnel to a depth of 10 mm by shortening the pulley system of the button. Next, the tibial fixation was performed using 4 all-suture anchors (MectaLock; Medacta). The anchors for fixation of the sMCL limb of the graft were placed at the anterior and posterior borders of the anatomic tibial sMCL insertion site. For the anteromedial limb, aiming to mimic the combined function of the dMCL and anteromedial retinaculum, the first anchor was placed 2 cm distal to the joint line and immediately in front of the sMCL limb. The second anchor was placed 20 mm anterior to the first anchor. The graft was sutured in a modified Krackow technique and fixed to the bone using the No. 2 suture of each anchor. Final tensioning was performed in 20° of flexion, by tightening the femoral adjustable button. Finally, the finished reconstruction was sutured to the posteromedial capsule and anteromedial retinaculum with No. 2 sutures (PowerSuture; Medacta) (Figure 3).

### Statistical Analysis

Extraction of knee kinematics from the raw data of simVITRO was performed using MATLAB (Version R2020a; MathWorks). Statistical analysis was performed using Prism (Version 10; GraphPad Software). The data were found to be normally distributed, utilizing histograms and the Shapiro-Wilk test. Means of single groups are presented with standard deviations. Mixed linear models with Geisser-Greenhouse correction were used to assess the main effects and interactions of each independent variable (cutting state and flexion angle). The dependent variables were valgus rotation (in degrees), ER (in degrees), ATT (in millimeters), and anteromedial translation (in millimeters). Pairwise comparisons with Dunn correction were used to compare the contribution of the states at different flexion angles. Multiple comparisons were performed against the native state, to refrain from unnecessary multiple comparisons. A *P* value <.05 was deemed to identify significant differences. Differences between means are presented as mean differences with corresponding 95% confidence intervals.

An a priori power analysis was performed using G*Power (Version 3.1).^17^ Based on means and standard deviations from a previous study on knee laxity,^46^ it was determined that a sample size of 8 knees would allow the identification of changes in translation/rotation of 2.0 ± 1.7 mm/deg (effect size, 1.2), with 80% power, at the significance level of *P* < .05. In total, 8 knees were used for final analysis because no specimen had to be excluded after testing.

## Results

Group means for the different performed movements are found in Appendix Table A1 (available in the online version of this article).

### Valgus Rotation

**Figure 4.**
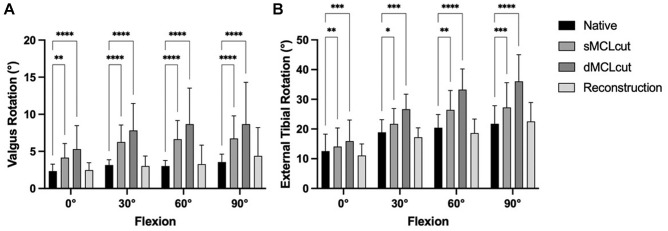
Influence of cutting and reconstruction in different flexion angles on (A) valgus rotation and (B) external tibial rotation. Deficiency of the superficial medial collateral ligament (sMCLcut) and deficiency of the deep medial collateral ligament (dMCLcut) resulted in significantly increased instability, which was redressable by the reconstruction (flat reconstruction of the sMCL and anteromedial corner). Multiple comparisons were performed against the native state. *P < .05; **P < .01; ***P < .001; ****P < .0001.

Cutting of the sMCL led to a significant increase in valgus rotation in all tested flexion angles (*P* < .05). Subsequent cutting of the dMCL led to a further increase in valgus rotation in all flexion angles (*P* < .05). The flat reconstruction of the sMCL and anteromedial corner reduced the valgus rotation to values not significantly different from those of the native knee (Figure 4A).

### External Tibial Rotation

Cutting of the sMCL led to a significant increase in ER in all tested flexion angles (*P* < .05). Subsequent cutting of the dMCL led to a further increase in ER in all flexion angles (*P* < .05). The flat reconstruction of the sMCL and anteromedial corner reduced ER to values not significantly different from those of the native knee (Figure 4B).

### Anterior Tibial Translation

**Figure 5.**
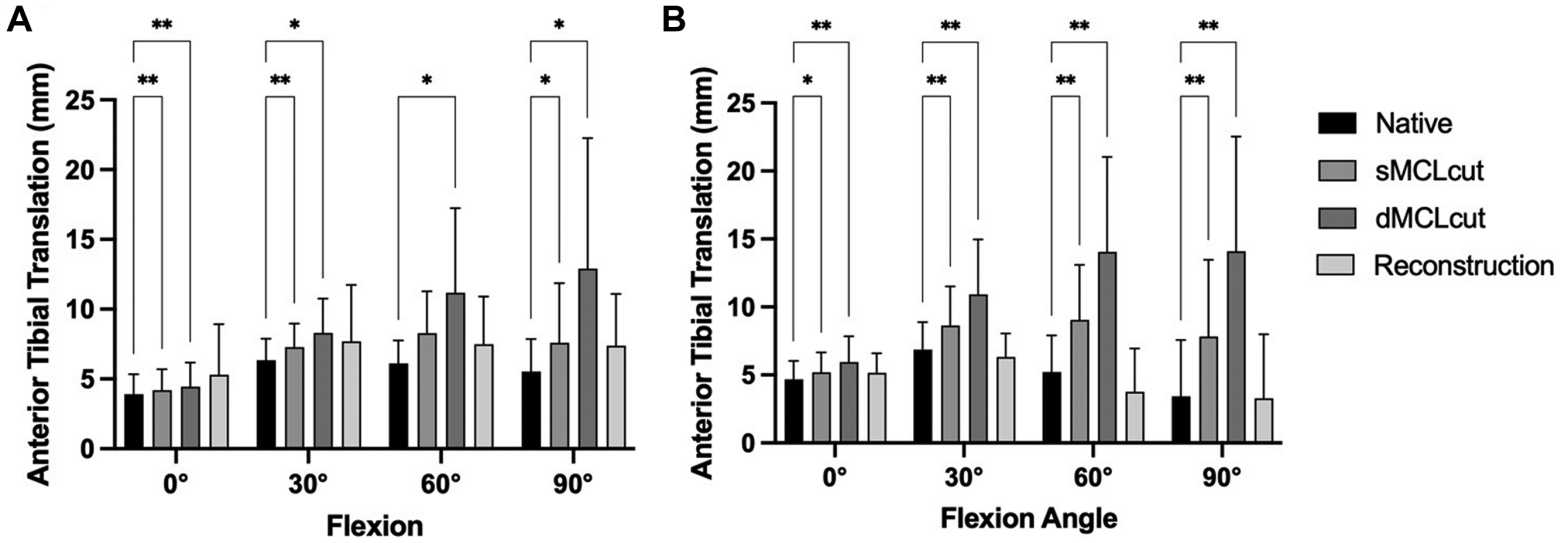
Influence of cutting and reconstruction in different flexion angles on (A) anterior tibial translation and (B) anteromedial translation. Deficiency of the superficial medial collateral ligament (sMCLcut) and deficiency of the deep medial collateral ligament (dMCLcut) resulted in significantly increased instability, which was redressable by the reconstruction (flat reconstruction of the sMCL and anteromedial corner). Multiple comparisons were performed against the native state. *P < .05; **P < .01.

Cutting of the sMCL led to a significant increase in ATT in 0°, 30°, and 90° of flexion (*P* < .05). Subsequent cutting of the dMCL led to a further significant increase in ATT in all flexion angles (*P* < .05). The flat reconstruction of the sMCL and anteromedial corner reduced ATT to values not significantly different from those of the native knee (Figure 5A).

### Anteromedial Tibial Translation (Slocum test)

Cutting of the sMCL led to a significant increase in ATT in all flexion angles (*P* < .05) (Figure 5B). Subsequent cutting of the dMCL led to a further significant increase in anteromedial tibial translation in all flexion angles (all *P* < .05). The flat reconstruction of the sMCL and anteromedial corner reduced the anteromedial tibial translation to values not significantly different from those of the native knee.

## Discussion

The most important finding of this study was that a flat reconstruction of the sMCL and anteromedial structures using a semitendinosus graft was able to restore native knee kinematics in knees with a deficient sMCL and dMCL in a cadaveric model. Furthermore, it was found that both an isolated, but more pronounced, insufficiency of the sMCL and a combined lesion of the sMCL and dMCL led to excess laxity in valgus rotation, ER, ATT, and anteromedial tibial rotation, even in ACL-intact knees.

Several biomechanical studies have investigated the restraining effect of structures on the medial side of the knee. While it was originally theorized that AMRI, marked by increased ATT as well as ER,^40^ is caused by a deficiency of the posterior oblique ligament,^33^ recent studies have found that the posterior oblique ligament has a negligible role in controlling AMRI.^4^ In contrast, the ACL, sMCL, and anteromedial structures (dMCL and AMC) have been highlighted as the crucial structures preventing excess AMRI.^4,22,25,37^ In a recent robotic biomechanical study utilizing the principle of superposition to determine the contribution of a structure on restraining knee movements, the ACL was found to be the primary restraint to AMRI between full extension and 30° of flexion.^22^ From 60° upward, the sMCL became the main contributor, restraining up to 36.8% of the AMRI. The dMCL and AMC resisted up to 23.1% of anteromedial translation, indicating a secondary role in controlling AMRI. On the other hand, the POL was not found to be a significant restrictor of AMRI. These findings are supported by other robotic biomechanical studies in human cadaveric knees, which found cutting of the dMCL led to markedly less AMRI compared with cutting of the sMCL, which was cut last in the sectioning protocol.^5,6,46^ In the present study, sectioning of the dMCL showed a comparably larger effect on knee instability, in comparison with the sMCL, which is in contrast to the previous studies. This observation is probably due to the fact that it was the last structure cut. Other studies have previously shown that with robotic force-controlled testing, the last structure to be cut typically leads to a major increase in instability.^25^ This underlines that both structures have to be deficient to lead to a large AMRI.

The present study is of relevance given the implications for clinical practice. Several techniques for reconstruction of the medial aspect of the knee have been described. These range from anteromedial tenodeses^9^ to single-bundle reconstructions^15,24^ to combined reconstructions of the sMCL with either the anteromedial aspect^9,45^ or posteromedial aspect.^26,27,42^ Furthermore, an isometric single-bundle reconstruction has been described.^12^ In previous biomechanical studies, it was found that isolated reconstructions of the sMCL are not able to fully restore native knee kinematics.^8,32^ A robotic biomechanical study on human cadaveric knee specimens showed that a single-bundle sMCL reconstruction was not able to restore anteromedial translation and ER and valgus rotation if the sMCL and dMCL were cut.^8^ In this study, reconstruction of the sMCL with a flat graft improved knee kinematics in comparison with a round graft. However, only an additional anteromedial reconstruction using a semitendinosus graft was able to fully restore the native knee kinematics. The finding of the aforementioned study were verified by a subsequent study, stating that in case of an anteromedial instability, a reconstruction of the sMCL and POL does not restore native knee kinematics, but only a combination of sMCL and dMCL.^32^ Furthermore, an isometric reconstruction of the anteromedial corner of the knee, utilizing high-strength tape, was described with promising biomechanical results.^11,38^ The present study underlines these previous findings, in that the presented flat reconstruction of the sMCL and anteromedial aspect of the knee are able to restore native knee kinematics and might therefore lead to favorable outcomes for patients with instabilities on the medial side of the knee. Furthermore, a previous study highlighted that different zones (anterior, intermediate, and posterior third) inside the sMCL perform different functions.^22^ Through use of a flat reconstruction, the whole surface of the sMCL can be reconstructed, ideally replicating the native anatomy without sacrificing strength of the tendon graft.^16^ Further advantages of the flat reconstruction might be improved tendon-to-bone healing because of the optimized tendon surface inside the bone tunnel.^49,50^ Future studies might investigate these possible biological advantages and compare the flat reconstruction against medial reconstruction techniques utilizing round tendon grafts.

This study is not without limitations. As with all biomechanical studies, this is a time-zero study not accounting for the postoperative healing process of the reconstruction. Cadaveric knee specimens of older age (mean age, 70.1 years) were used, which might not necessarily reflect the clinical reality of bone and soft tissue quality. For tibial fixation, knotless suture anchors were used in this study. However, numerous other fixation devices, including knotless anchors or staples are available to create flat grafts.^14,18^ How these fixation modalities might influence the performance of the described technique could not be evaluated. A major limitation of this study was that no second reconstruction technique was performed as a comparison to the flat reconstruction. The rationale for this was that creation of a flat femoral tunnel, as well as placing multiple suture anchors in the proximal tibia, might compromise the fixation of a subsequently performed round reconstruction toward inferior results. In the present study, the ACL was left intact, even though injuries to the medial side frequently occur concomitantly with ACL injuries. The effect of cutting the medial structures in the ACL-injured state was previously investigated.^4,46^ In the present study, the ACL was left intact to simulate an optimal ACL reconstruction, as in previous studies.^34^ Leaving the ACL intact was done to reduce the confounding effect of different ACL reconstruction techniques on the results. Finally, the flat reconstruction showed no significant differences from the native state. This lack of a difference, however, does not mean that the reconstructed and native states are equivalent, because this study was only powered to test for differences between states, but underpowered to determine the equivalence of states.^21^ However, means of the reconstructed and native states are similar (see Figures 4 and 5), so that differences between the groups that might show with a higher sample size might be small and of questionable clinical relevance.

## Conclusion

Insufficiency of the sMCL and dMCL led to excess valgus rotation, ER, ATT, and anteromedial tibial translation. A combined flat reconstruction of the sMCL and the anteromedial aspect restored this excess laxity to values not significantly different from those of the native knee.

## Supplemental Material

sj-docx-1-ajs-10.1177_03635465241280984 – Supplemental material for A Flat Reconstruction of the Medial Collateral Ligament and Anteromedial Structures Restores Native Knee Kinematics: A Biomechanical Robotic Investigation Supplemental material, sj-docx-1-ajs-10.1177_03635465241280984 for A Flat Reconstruction of the Medial Collateral Ligament and Anteromedial Structures Restores Native Knee Kinematics: A Biomechanical Robotic Investigation by Adrian Deichsel, Christian Peez, Michael J. Raschke, Alina Albert, Mirco Herbort, Christoph Kittl, Christian Fink and Elmar Herbst in The American Journal of Sports Medicine
