# Supplementary material for: A Flat Reconstruction of the Medial Collateral Ligament and Anteromedial Structures Restores Native Knee Kinematics: A Biomechanical Robotic Investigation
Source: Am J Sports Med. 2024 Oct 3;52(13):3306–13. doi: 10.1177/03635465241280984 (PMC11542325; doi:10.1177/03635465241280984)
Supplement: sj-docx-1-ajs-10.1177_03635465241280984 – Supplemental material for A Flat Reconstruction of the Medial Collateral Ligament and Anteromedial Structures Restores Native Knee Kinematics: A Biomechanical Robotic Investigation [file sj-docx-1-ajs-10.1177_03635465241280984.docx]

**A Flat Reconstruction of the Medial Collateral Ligament and Anteromedial Structures Restores Native Knee Kinematics
- A Biomechanical Robotic Investigation**

**APPENDIX**

**Table A1:** Influence of cutting and reconstruction in different flexion angles on knee kinematics. sMCLcut = deficiency of the superficial medial collateral ligament; dMCLcut = deficiency of the deep medial collateral ligament; Reconstruction = Flat reconstruction of the sMCL and anteromedial corner. Multiple comparisons were performed against the native state; * = P < 0.05; ** = P < 0.01; *** = P < 0.001; **** = P < 0.0001.

|  | **Flexion** | **Native** | **sMCLcut** | **dMCLcut** | **Reconstruction** |
| --- | --- | --- | --- | --- | --- |
| Valgus rotation (°) |  |  |  |  |  |
|  | 0° | 2.3 ± 0.9 | 4.2 ± 1.9 (****) | 5.3 ± 3.2 (******) | 2.5 ± 1.0 (P = 0.99) |
|  | 30° | 3.2 ± 0.7 | 6.3 ± 2.3 (******) | 7.8 ± 3.6 (******) | 3.0 ± 1.4 (P = 0.99) |
|  | 60° | 3.0 ± 0.7 | 6.6 ± 2.5 (******) | 8.7 ± 4.9 (******) | 3.3 ± 2.6 (P = 0.95) |
|  | 90° | 3.6 ± 1.1 | 6.7 ± 3.1 (******) | 8.7 ± 5.6 (******) | 4.4 ± 3.8 (P = 0.32) |
| External tibial Rotation (°) |  | | | | |
|  | 0° | 12.6 ± 5.7 | 14.1 ± 6.3 (****) | 15.9 ± 7.1 (*****) | 11.1 ± 3.9 (P = 0.18) |
|  | 30° | 18.9 ± 4.2 | 21.7 ± 5.2 (***) | 26.7 ± 5.1 (*****) | 17.2 ± 3.2 (P = 0.12) |
|  | 60° | 20.4 ± 4.5 | 26.4 ± 6.5 (****) | 33.3 ± 7.0 (******) | 18.6 ± 4.7 (P = 0.10) |
|  | 90° | 21.8 ± 6.1 | 27.3 ± 8.3 (*****) | 36.1 ± 8.9 (******) | 22.6 ± 6.4 (P = 0.76) |
| Anterior tibial translation (mm) |  | | | | |
|  | 0° | 3.9 ± 1.4 | 4.2 ± 1.5 (****) | 4.4 ± 1.7 (****) | 5.3 ± 3.6 (P = 0.17) |
|  | 30° | 6.3 ± 1.5 | 7.3 ± 1.7 (****) | 8.3 ± 2.5 (***) | 7.7 ± 4.0 (P = 0.24) |
|  | 60° | 6.1 ± 1.7 | 8.3 ± 3.0 | 11.2 ± 6.1 (***) | 7.5 ± 3.4 (P = 0.19) |
|  | 90° | 5.5 ± 2.3 | 7.6 ± 4.3 (***) | 12.9 ± 9.3 (***) | 7.4 ± 3.7 (P = 0.16) |
| Anteromedial tibial translation (mm) |  | | | | |
|  | 0° | 4.7 ± 1.4 | 5.2 ± 1.5 (***) | 6.0 ± 1.9 (****) | 5.2 ± 1.4 (P = 0.27) |
|  | 30° | 6.9 ± 2.0 | 8.6 ± 2.9 (****) | 10.9 ± 4.0 (****) | 6.3 ± 1.7 (P = 0.27) |
|  | 60° | 5.2 ± 2.7 | 9.0 ± 4.1 (****) | 14.1 ± 7.0 (****) | 3.8 ± 3.2 (P = 0.31) |
|  | 90° | 3.4 ± 4.1 | 7.8 ± 5.7 (****) | 14.1 ± 8.4 (****) | 3.3 ± 4.7 (P = 0.99) |
